# Virological response and predictive factors for antiviral treatment in chronic HBV-related liver disease with low ALT and high HBV DNA

**DOI:** 10.3389/fimmu.2025.1556547

**Published:** 2025-02-26

**Authors:** Lei Ma, Yan Li, Lihan Weng, Huichun Xing

**Affiliations:** ^1^ Center of Liver Diseases Division 3, Beijing Ditan Hospital, Capital Medical University, Beijing, China; ^2^ Center of Liver Diseases Division 3, Peking University Ditan Teaching Hospital, Beijing, China

**Keywords:** chronic HBV-related liver disease, antiviral treatment, virological responses, low ALT levels, high viral load

## Abstract

**Objective:**

To investigate virological response and predictive factors for antiviral treatment in chronic HBV patients with low ALT and high HBV DNA.

**Methods:**

A retrospective study grouped chronic HBV patients by baseline ALT: ALT > 80 U/L (significantly elevated group, SAG), 40-80 U/L (mildly elevated group, MAG), and ≤ 40 U/L (normal group, NG). Inverse probability treatment weighting balanced confounding factors. Complete virological response (CVR, HBV DNA < 20 IU/mL) and partial virological response (PVR, HBV DNA ≥ 20 IU/mL) were defined. NG subgroup analyses were performed using baseline ALT (cutoff: 30 U/L for males, 19 U/L for females), HBV DNA (cutoff: 7.21 Log_10_ IU/mL), and Aspartate Aminotransferase to Platelet Ratio Index (cutoff: 0.32). Cox regression identified factors predicting CVR at week 48.

**Results:**

After IPTW, the number of patients in the NG, MAG, and SAG groups was 92, 141, and 284, and the CVR rates at week 48 were 38.05%, 55.26%, and 7analyses 3.32% respectively (*p* < 0.0001). Weighted Kaplan-Meier analysis showed that the NG group had the lowest probability of achieving CVR at week 48 (*p* < 0.0001). Particularly, in the NG group, the high-normal ALT subgroup had a higher CVR rate (56.34% (40/71)) than the low-normal ALT subgroup (29.73% (11/37), *p* = 0.0103), similar to that of the MAG group (*p* = 0.9871). The low-HBV DNA (82.46% (47/57)) and high-APRI subgroup (63.79% (37/58)) had higher CVR rates than the high-HBV DNA (7.84% (4/51)) and low-APRI subgroup (28% (14/50)) respectively. High HBV DNA and low ALT patients in NG had a CVR of 0% (0/18). Cox regression identified baseline ALT ≤ 30 U/L (males) or ALT ≤ 19 U/L (females), HBV DNA > 7.21 Log_10_ IU/mL, HBeAg positive state, APRI < 0.32, and a decrease in HBV DNA < 3.49 Log_10_ IU/mL at 12 weeks as independent adverse predictors of CVR.

**Conclusion:**

The NG group has lower CVR, but the high-normal ALT subgroup performs similarly to MAG. High HBV DNA and low ALT significantly reduce CVR. Key adverse predictors include low ALT, high HBV DNA, HBeAg positivity, low APRI, and suboptimal viral reduction at 12 weeks.

## 1 Introduction

Hepatitis B virus (HBV) infection poses a significant global public health challenge and is a leading cause of liver disease. According to the epidemiological report released by the WHO, approximately 296 million people are chronically infected with HBV worldwide (1). Persistent HBV DNA replication drives disease progression, increasing the risk of complications such as cirrhosis, liver failure, and hepatocellular carcinoma (HCC). However, the rates of diagnosis (13%) and treatment (3%) are significantly lower than the WHO targets (90% for diagnosis and 80% for treatment) (2). To mitigate these risks and meet the WHO’s goal of eliminating viral hepatitis as a public health threat by 2030, regional guidelines advocate for early and more active antiviral therapy. This approach effectively expands the treatment population, which is promising in suppressing viral replication and promoting a virological response, thereby alleviating hepatocellular inflammation, necrosis, and fibrosis, and helping to slow or even reverse disease progression and prevent severe complications. However, the viral response of NAs in patients with a high viral load and low ALT still requires thorough study.

Alanine aminotransferase (ALT), a marker of liver injury, reflects hepatic immune status, which is associated with leukocyte infiltration and necrotizing inflammation. It is widely used to guide the initiation of antiviral therapy. However, studies have indicated that significant histological changes occur in about one-third of chronic hepatitis B (CHB) patients with normal ALT levels (3). Moreover, a study has shown that even in HBeAg-negative patients with ALT levels ranging from 21 to 40 U/L, more than 50% still exhibit hepatic necroinflammation (4). Across all disease stages, chronic HBV infection is characterized by dysregulation of immune gene expression and metabolic pathways (5). Studies by Kim and Choi in South Korea have shown that untreated CHB patients with normal ALT levels had a significantly higher risk of liver cancer, transplantation, or death compared to those with elevated ALT who received treatment (6, 7). Patients with normal ALT, which is conventionally regarded as safe, still pose a significant risk for severe liver lesions and adverse outcomes. Current guidelines recommend initiating antiviral therapy once ALT levels exceed the normal threshold. The latest WHO guidelines (2024) (8) and the expert consensus (2021) (9) propose further lowering the antiviral thresholds to 30 U/L for males and 19 U/L for females, aiming to expand treatment coverage and reduce liver-related morbidity and mortality. However, studies on virological response to antiviral therapy in HBV-related liver disease patients with normal ALT are limited and inconsistent due to differences in ethnicity, baseline ALT thresholds, viral load, and immune status. Based on China’s 2022 Guidelines and our hospital’s laboratory standards, which use 40 U/L as the ALT normal threshold, this study aimed to evaluate the virological response to antiviral therapy in chronic HBV-related liver disease patients with low ALT levels and high viral loads. Additionally, the antiviral efficacy across subgroups categorized by ALT levels, HBV DNA load, and Aspartate Aminotransferase to Platelet Ratio Index (APRI) scores within the normal ALT group, as well as the factors influencing virological response, were also explored.

## 2 Methods

### 2.1 Study design

This retrospective cohort study selected treatment-naïve patients with chronic HBV-related liver disease, including CHB, cirrhosis, and liver failure, from Beijing Ditan Hospital, Capital Medical University between April 1, 2020, and January 31, 2022. Antiviral therapy was initiated with a single nucleos(t)ide analog (NAs), Tenofovir Alafenamide (TAF, 25 mg/day), Tenofovir Disoproxil Fumarate (TDF, 300 mg/day), or Entecavir (ETV, 0.5 mg/day)). The inclusion criteria were as follows (1): age >18 years (2), HBsAg positive for >6 months (3), HBV DNA ≥ 10^5^ IU/mL (4), no prior NAs or interferon therapy, or previous treatment discontinued for >1 year due to self-interruption (5), antiviral therapy limited to ETV, TDF, or TAF (6), completion of 48 weeks of follow-up after therapy initiation. Patients were excluded if they had other viral hepatitis, drug-induced or autoimmune liver disease, alcoholic liver disease, human immunodeficiency virus (HIV), pregnancy, HCC, or malignancies, were lost to follow-up, combined additional antivirals or interferon, discontinued treatment within 48 weeks, or had severe systemic comorbidities.

The enrolled patients were divided into three groups based on baseline ALT levels: ALT >80 U/L as the significantly elevated ALT group (SAG), 40 < ALT ≤80 U/L as the mildly elevated ALT group (MAG), and ALT ≤40 U/L as the normal ALT group (NG). Subgroup analyses were further performed within the NG group. Based on baseline ALT levels, HBV DNA loads, and APRI scores as follows (**Supplementary Table 1**).

Males with 30 < ALT ≤ 40 U/L or females with 19 < ALT ≤ 40 U/L were defined as the high-normal ALT subgroup (HNA), while males with ALT ≤30 U/L or females with ALT ≤19 U/L as the low-normal ALT subgroup (LNA). The optimal cutoff for baseline HBV DNA in the NG group was determined using ROC curve analysis and the Youden index to distinguish the likelihood of virological response at week 48 of antiviral treatment. Patients with values above the cutoff were classified as the high-HBV DNA subgroup (N-H-HBV), while those below as the low-HBV DNA subgroup (N-L-HBV). Similarly, the optimal baseline APRI score in the NG group was determined, with patients above the cutoff classified as the high-APRI subgroup (N-H-APRI) and those below as the low-APRI subgroup (N-LAPRI). Baseline data, including demographics, virological parameters, liver function, blood lipids, alpha-fetoprotein (AFP), liver stiffness values, abdominal imaging, and follow-up virological data at weeks 4, 12, 24, and 48 were collected for all groups. According to the HBV DNA levels at week 48, patients were classified into the complete virological response group (CVR, HBV DNA <20 IU/mL) and partial virological response (PVR, regular antiviral treatment with HBV DNA remaining above 20 IU/mL but below baseline) at week 48. Univariate and multivariate Cox regression analyses were conducted to identify predictors of CVR at week 48 of antiviral therapy.

The Institutional Review Committee of Beijing Ditan Hospital, Capital Medical University approved this study. As retrospective data with anonymized patient information were used, the requirement for informed consent was waived. All procedures adhered to the Declaration of Helsinki.

### 2.2 Study outcomes

The primary endpoint of this study is the CVR rate at week 48 in the normal ALT group. Secondary endpoints encompass (1): comparison of the CVR rate at week 48 among different ALT level groups (2); the factors influencing the CVR rate in patients with normal ALT; and (3) the HBeAg seroconversion rate and HBsAg decline at each time point during follow-up among different ALT level groups.

### 2.3 Statistical analysis

Continuous variables were depicted as mean ± SD, or median (IQR) and evaluated using Wilcoxon’s rank-sum test. Categorical variables were presented as percentages and analyzed through Chi-square tests. Censored follow-up refers to cases where a CVR was not achieved by week 48 of antiviral therapy.

Inverse probability of treatment weighting (IPTW) was employed to weight and match age, gender, diabetes history, drinking habits, HBV DNA levels, HBeAg status, HBsAg levels, triglycerides, AFP, and liver stiffness values, ensuring comparable baseline characteristics among the three groups and minimize potential confounders that might affect antiviral efficacy. The balance among the three groups was evaluated using p-values and standardized mean differences (SMD), with *p* > 0.05 or SMD < 0.1 indicating good balance. The Kaplan-Meier method and log-rank test were employed to compare the proportion of patients with a CVR at week 48. Univariate and multivariate Cox proportional hazards regression analyses were conducted to identify predictors influencing virological response at week 48. Variables with clinical relevance or *p* < 0.05 in univariate analysis were incorporated in the multivariate Cox model. Variables were carefully selected to maintain the simplicity of the final model. All authors had access to the study data, and the final manuscript was reviewed and approved. Statistical analysis was carried out using R (version 4.3.2, R Foundation for Statistical Computing, Beijing, China), with a two-sided p-value < 0.05 regarded as statistically significant.

## 3 Results

### 3.1 Characteristics of the total cohort

**Figure 1:**
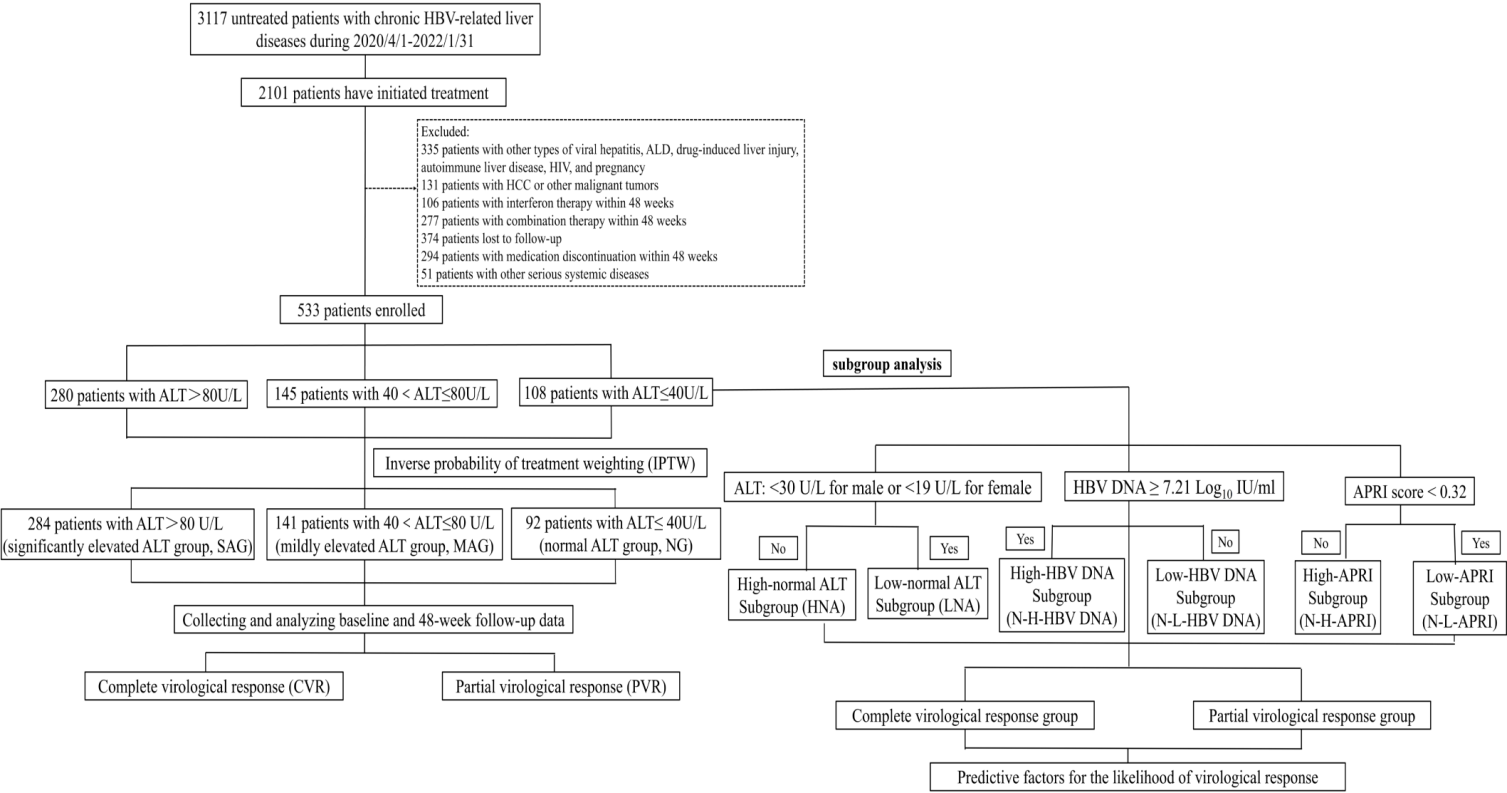
Flowchart of patient selection. ALD, alcoholic liver disease; ALT, alanine aminotransferase; HCC, hepatocellular carcinoma; HIV, human immunodeficiency virus.

A total of 3117 patients with treatment-naïve chronic HBV-related liver disease were selected, and 2101 **initiated** antiviral treatment. Based on the inclusion criteria, 533 patients were finally enrolled in this analysis (**Figure 1**). Among them, 108, 145, and 280 patients were classified into the NG, MAG, and SAG groups, respectively. At baseline, the mean age of the total cohort was 39 years; 323 (60.6%) were male, 36 (6.8%) had hypertension, 15 (2.8%) had diabetes mellitus, 53 (9.9%) had drinking habits, and 96 (18%) had metabolic dysfunction-associated steatotic liver disease (MASLD). Patients in the NG group were the oldest compared with those in the MAG and SAG groups (40.6 vs 40.4 vs 37.8 years, *p* = 0.008), and had the lowest HBV DNA levels (6.93 vs 7.01 vs 7.3 Log_10_ IU/mL, *p* = 0.004), and the lowest liver stiffness values (9.47 vs 10.9 vs 16.4 kPa, *p* < 0.001) at the initiation of antiviral therapy. The proportions of patients receiving ETV, TDF, and TAF for antiviral treatment were similar across the three groups. Additionally, within the NG group, there were no significant differences in the distribution of ETV, TDF, and TAF usage among its subgroups. Detailed baseline characteristics are presented in **Table 1**.

**Table 1:** Baseline characteristics of treatment-naïve chronic HBV-related liver disease patients stratified by ALT levels.

	Before IPTW				After IPTW				
	ALT>80U/L (SAG) n=280	40<ALT ≤ 80U/L (MAG) n=145	ALT ≤ 40U/L (NG) n=108	*p*	ALT>80U/L (SAG) n=284	40<ALT ≤ 80U/L (MAG) n=141	ALT ≤ 40U/L (NG) n=92	*p*	SMD
Age (years)	37.8 ± 9.78	40.4 ± 10.2	40.6 ± 10.9	0.008	39.74 ± 11.46	39.83 ± 10.37	40.38 ± 10.53	0.917	0.04
Male sex (%)	188 (67.1%)	95 (65.5%)	40 (37.0%)	<0.001	174.2 (61.3)	82.4 (58.5)	48.3 (52.5)	0.425	0.119
Hypertension (%)	15 (5.36%)	14 (9.66%)	7 (6.48%)	0.244	16.4 (5.8)	13.5 (9.6)	8.5 (9.2)	0.504	0.097
Diabetes (%)	6 (2.14%)	7 (4.83%)	2 (1.85%)	0.243	9.7 (3.4)	7.9 (5.6)	1.6 (1.7)	0.449	0.141
Alcohol habit (%)	38 (13.6%)	12 (8.28%)	3 (2.78%)	0.005	27.6 (9.7)	11.5 (8.2)	4.4 (4.7)	0.459	0.129
ALT (U/L)	341 ± 359	56.3 ± 11.3	27.8 ± 8.28	<0.001	294.72 ± 315.57	55.83 ± 11.46	29.20 ± 7.65	<0.001	1.665
AST (U/L)	199 ± 239	41.0 ± 16.0	26.8 ± 10.8	<0.001	173.74 ± 207.05	43.91 ± 17.41	29.82 ± 14.15	<0.001	0.917
Tbil (umol/L)	30.0 ± 42.2	16.2 ± 7.90	15.0 ± 7.25	<0.001	24.80 ± 32.56	17.65 ± 10.26	16.56 ± 8.58	<0.001	0.253
ALB (g/L)	43.7 ± 4.89	45.0 ± 5.43	44.4 ± 5.22	0.060	44.18 ± 4.35	43.87 ± 6.37	43.13 ± 6.65	0.558	0.118
GGT (U/L)	86.8 ± 89.3	42.2 ± 47.0	22.5 ± 20.2	<0.001	74.08 ± 76.52	43.05 ± 44.21	27.61 ± 23.24	<0.001	0.585
ALP (U/L)	82.6 ± 32.0	72.2 ± 21.1	69.3 ± 22.9	<0.001	79.96 ± 30.36	74.88 ± 26.61	73.40 ± 23.81	0.165	0.159
HBV DNA (Log_10_ IU/mL)	7.30 ± 1.03	7.01 ± 1.2	6.93 ± 1.26	0.004	7.13 ± 1.09	7.14 ± 1.15	7.19 ± 1.18	0.917	0.036
HBsAg (Log_10_ IU/mL)	3.77 ± 0.70	3.93 ± 0.65	3.93 ± 0.77	0.035	3.85 ± 0.69	3.86 ± 0.67	3.91 ± 0.81	0.891	0.047
HBeAg positive (%)	208 (74.3%)	108 (74.5%)	84 (77.8%)	0.763	216 (76.1)	106 (75.2)	75 (81.5)	0.477	0.108
PLT (×109/L)	194 ± 61.3	195 ± 64.4	206 ± 62.0	0.206	196.08 ± 57.28	184.23 ± 66.72	193.32 ± 68.23	0.272	0.123
TCHO (mmol/L)	4.47 ± 0.91	4.72 ± 0.90	4.55 ± 0.88	0.020	4.53 ± 0.91	4.57 ± 0.97	4.47 ± 0.92	0.807	0.07
TG (mmol/L)	1.30 ± 0.65	1.20 ± 0.60	0.98 ± 0.45	<0.001	1.21 ± 0.58	1.15 ± 0.63	1.08 ± 0.50	0.232	0.145
Cr (umol/L)	70.3 ± 14.5	71.5 ± 13.6	64.1 ± 14.1	<0.001	69.22 ± 14.82	69.78 ± 13.86	66.45 ± 14.48	0.277	0.154
Glu (mmol/L)	5.55 ± 1.35	5.65 ± 1.00	5.53 ± 0.68	0.631	5.64 ± 1.43	5.56 ± 0.89	5.52 ± 0.72	0.728	0.073
INR	1.17 ± 0.23	1.15 ± 0.19	1.13 ± 0.16	0.114	1.15 ± 0.18	1.18 ± 0.22	1.17 ± 0.24	0.304	0.118
AFP (ng/ml)	56.2 ± 185	11.2 ± 42.4	5.05 ± 8.32	<0.001	34.02 ± 136.35	23.21 ± 77.34	9.47 ± 14.80	0.001	0.199
CEA	2.55 ± 1.37	2.59 ± 1.41	2.21 ± 1.03	0.047	2.45 ± 1.28	2.57 ± 1.42	2.51 ± 1.33	0.753	0.059
LSM (kPa)	16.4 ± 12.8	10.9 ± 7.9	9.47 ± 8.05	<0.001	13.97 ± 10.69	12.86 ± 9.91	12.38 ± 10.80	0.498	0.1
MASLD (%)	48 (17.1%)	32 (22.1%)	16 (14.8%)	0.286	44 (15.5%)	25 (17.7%)	14 (15.2%)	0.806	0.047
Ascites (%)	16 (5.71%)	9 (6.21%)	6 (5.56%)	0.971	11.0 (3.9)	16.4 (11.6)	8.0 (8.7)	0.056	0.197
Splenomegaly (%)	60 (21.4%)	30 (20.7%)	17 (15.7%)	0.445	50.6 (17.8)	35.5 (25.2)	18.9 (20.5)	0.332	0.121
Drug_type ETV	68 (24.3%)	34 (23.4%)	29 (26.9%)	0.511	71 (25%)	34 (24.1%)	26 (28.3%)	0.511	0.168
TDF	82 (29.3%)	40 (27.6%)	22 (20.4%)		78 (27.5%)	39 (27.7%)	16 (17.4%)		
TAF	130 (46.4%)	71 (49%)	57 (52.8%)		135 (47.5%)	68 (48.2%)	50 (54.3%)		

Values presented as n (%), mean ± SD.

AFP, alpha-fetoprotein; ALB, albumin; ALP, alkaline phosphatase; ALT, alanine aminotransferase; AST, aspartate aminotransferase; CEA, carcinoembryonic antigen; Cr, creatinine; GGT, gamma-glutamyl transferase; Glu, glucose; HBeAg, hepatitis B e antigen; HBsAg, hepatitis B surface antigen; INR, international normalized ratio; LSM, liver stiffness measurement; MASLD, metabolic dysfunction-associated steatotic liver disease; PLT, platelets; TCHO, total cholesterol; TG, triglycerides; Tbil, total bilirubin.

To balance the baseline characteristics among the three groups, IPTW was applied, resulting in an adjusted cohort of 517 patients, consisting of 92, 141, and 284 patients in the NG, MAG, and SAG groups, respectively. Except for liver function indicators (ALT, AST, TB, GGT), the indicators were well-balanced with no significant differences among the three groups (**Table 1**; **Supplementary Figure 1**).

### 3.2 Comparison of HBV DNA level changes among the three groups

**Figure 2:**
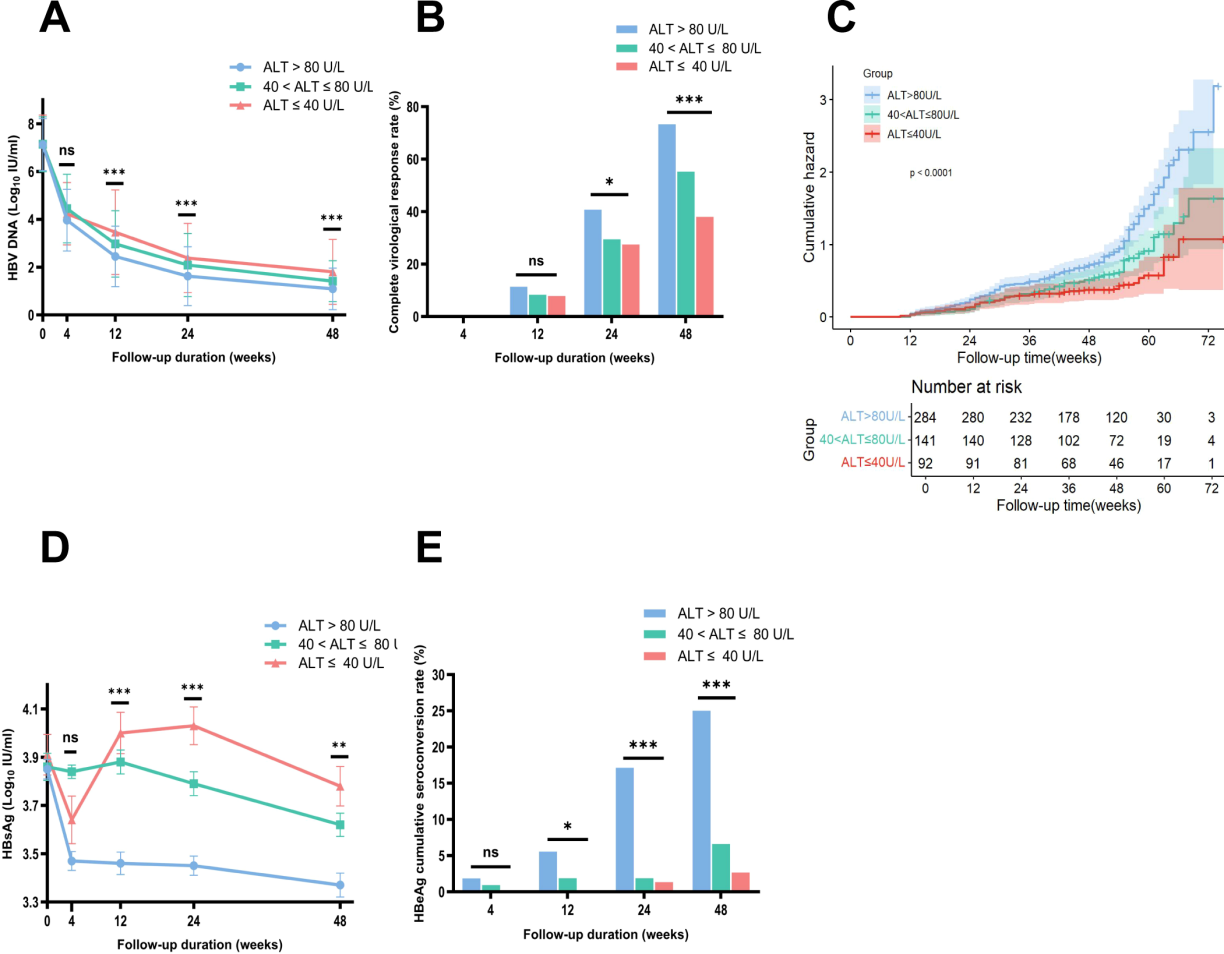
Comparison of HBV DNA changes, complete virological response rates, HBsAg loss, and HBeAg seroconversion rates among three groups during follow-up, with Kaplan-Meier analysis after IPTW adjustment. **(A)** Comparison of changes in HBV DNA levels during follow-up after IPTW. **(B)** Comparison of complete virological response rates at each time point among three groups after IPTW. **(C)** Weighted Kaplan-Meier analysis showed that the probability of achieving a CVR within 48 weeks was the lowest in the NG group among the three groups. **(D)** Comparison of changes in HBsAg levels during follow-up after IPTW. **(E)** Comparison of HBeAg seroconversion rates at each time point among three groups after IPTW. ALT, alanine aminotransferase; HBeAg, hepatitis B e antigen; HBsAg, hepatitis B surface antigen. Error bars represent the standard deviation. *p<0.05, **p<0.01, ***p<0.001. ns, no statistically significant difference.

After IPTW adjustment, the weighted baseline HBV DNA levels of the three groups (NG, MAG, SAG) were well-matched and balanced (7.19 vs 7.14 vs 7.13 Log_10_ IU/mL respectively, *p* = 0.917, SMD = 0.036). Following antiviral therapy, HBV DNA levels in all three groups exhibited a downward trend, with the steepest decline occurring in the first four weeks, followed by a gradual deceleration. At week 48, the mean HBV DNA level in the NG group was the highest compared with those in the MAG and SAG groups (1.80 vs 1.41 vs 1.09 Log_10_ IU/mL, *p* < 0.001) (**Figure 2A**). The mean HBV DNA levels were also comparable at weeks 12 and 24 (at week 12: 3.46 vs 2.97 vs 2.45 Log_10_ IU/mL, *p* < 0.001; at week 24: 2.38 vs 2.09 vs 1.62 Log_10_ IU/mL, *p* = 0.001, respectively).

### 3.3 Comparison of virological response rates and Kaplan-Meier curves among the three groups

Before IPTW, the CVR rate at week 48 for the total cohort was 64.35% (343/533), with rates of 47.22% (51/108), 55.17% (80/145), and 75.71% (212/280) in the NG, MAG, and SAG groups, respectively. After IPTW adjustment, the overall CVR rate at week 48 was 62.12% (321/517), with rates of 38.05% (35/92), 55.26% (78/141), and 73.32% (208/284) in the NG, MAG, and SAG groups respectively, showing a significant difference (*p* < 0.0001) (**Figure 2B**). The difference in CVR rates was also evident at week 24, with rates of 27.51% (25/92), 29.44% (42/141), and 40.76% (116/284) in the NG, MAG, and SAG groups, respectively (*p* = 0.015). Weighted Kaplan-Meier analysis showed that the probability of achieving a CVR within 48 weeks was lowest in the NG group among the three groups (*p* < 0.0001) (**Figure 2C**).

### 3.4 Comparison of HBsAg decline and HBeAg seroconversion rates among the three groups

At baseline, the mean HBsAg level in the NG group was the highest among the three groups. After IPTW, the baseline HBsAg levels were balanced and comparable across the NG, MAG, and SAG groups (3.91 vs 3.86 vs 3.85 Log_10_ IU/mL, *p* = 0.891, SMD = 0.047). After antiviral therapy, HBsAg levels in all groups showed a downward trend. At week 48, the mean HBsAg level in the NG group was the highest compared with those in the MAG and SAG groups (3.78 vs 3.62 vs 3.37 Log_10_ IU/mL, *p* = 0.002) (**Figure 2D**). Additionally, the mean HBsAg levels in the NG group were also the highest at both week 12 and week 24 (at week 12: 4.00 vs 3.88 vs 3.46 Log_10_ IU/mL, *p* < 0.001; at week 24: 4.03 vs 3.79 vs 3.45 Log_10_ IU/mL, *p* < 0.001).

After IPTW, 397 patients (76.79%) in the total cohort were HBeAg positive at baseline. Among them, 75 (81.5%), 106 (75.2%), and 216 (76.1%) were in the NG, MAG, and SAG groups, respectively (*p* = 0.477). At week 48, the HBeAg seroconversion rate in the NG group was the lowest compared with those in the MAG and SAG groups (2.67% (2/75) vs 6.60% (7/106) vs 25% (54/216), *p* < 0.001) (**Figure 2E**). Similar results **for** HBeAg seroconversion rates were also observed at weeks 12 and 24 (at week 12: 0% (0/75) vs 1.89% (2/106) vs 5.56% (12/216), *p* = 0.045; at week 24: 1.33% (1/75) vs 1.89% (2/106) vs 17.13% (37/216), *p* < 0.001).

### 3.5 Subgroup analysis of the normal ALT group

#### 3.5.1 Stratified analysis by ALT levels

**Figure 3:**
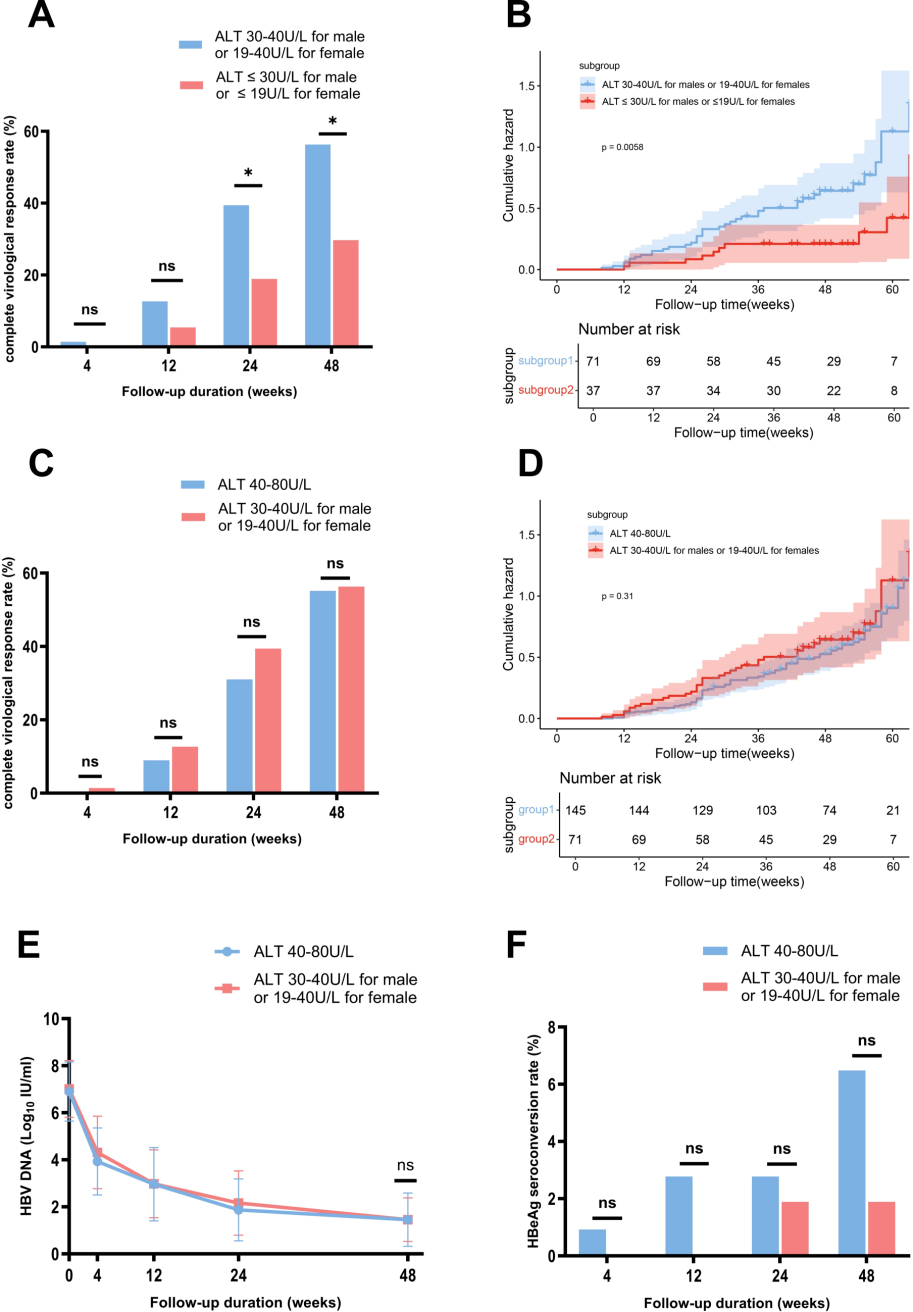
Comparison of HBV DNA changes, complete virological response rates, and HBeAg seroconversion rates between the subgroups stratified by ALT Levels, with Kaplan-Meier analysis. **(A)** Comparison of complete virological response rates between the HNA and LNA subgroups. **(B)** Kaplan-Meier survival analysis showed a higher likelihood of achieving a complete virological response in the HNA subgroup than in the LNA subgroup over 48 weeks of therapy. **(C)** Comparison of complete virological response rates between the HNA subgroup and MAG group. **(D)** Kaplan-Meier survival analysis showed a similar likelihood of achieving a complete virological response at week 48 between the HNA subgroup and MAG group. **(E)** Comparison of changes in HBV DNA levels between the HNA subgroup and MAG group. **(F)** Comparison of HBeAg seroconversion rates between the HNA subgroup and MAG group. ALT, alanine aminotransferase; HBeAg, hepatitis B e antigen; HNA, high normal ALT subgroup; MAG, mildly elevated ALT group. ns, no statistically significant difference. Error bars represent the standard deviation. *p<0.05.

Based on baseline ALT levels, we further stratified patients in the NG group into two subgroups: the HNA subgroup (30 < ALT ≤ 40 U/L for males or 19 < ALT ≤ 40 U/L for females) with 71 patients, and the LNA subgroup (ALT ≤ 30 U/L for males or ALT ≤ 19 U/L for females) with 37 patients. **Table 2** summarizes the baseline characteristics of the two subgroups. At week 48, the CVR rate was significantly higher in the HNA subgroup (56.34% (40/71)) compared to the LNA subgroup (29.73% (11/37), *p* = 0.0103) (**Figure 3A**). Kaplan-Meier analysis further revealed a higher likelihood of achieving a CVR in the HNA subgroup than in the LNA subgroup over 48 weeks of therapy (*p* = 0.0058) (**Figure 3B**).

**Table 2:** Baseline characteristics of the normal ALT group stratified by different clinical indicators.

	High-normal ALT subgroup (HNA) n=71	Low-normal ALT subgroup (LNA) n=37	*p*	Low-HBV DNA subgroup (N-L-HBV) n=57	High-HBV DNA subgroup (N-H-HBV) n=51	*p*	High-APRI subgroup (N-H-APRI) n=58	Low-APRI subgroup (N-L-APRI) n=50	*p*
Age (years)	40.3 ± 10.9	41.3 ± 11.0	0.649	43.8 ± 11.4	37.1 ± 9.15	0.001	43.8 ± 11.4	36.8 ± 9.0	0.001
Male sex (%)	21 (29.6%)	19 (51.4%)	0.044	19 (33.3%)	21 (41.2%)	0.520	22 (37.9%)	18 (36.0%)	0.994
Hypertension (%)	5 (7.04%)	2 (5.41%)	1	5 (8.77%)	2 (3.92%)	0.443	7 (12.1%)	0 (0.00%)	0.014
Diabetes (%)	2 (2.82%)	0 (0.00%)	0.545	2 (3.51%)	0 (0.00%)	0.497	2 (3.45%)	0 (0.00%)	0.498
Alcohol habit (%)	2 (2.82%)	1 (2.70%)	1	3 (5.26%)	0 (0.00%)	0.245	3 (5.17%)	0 (0.00%)	0.247
ALT (U/L)	32.0 ± 6.10	19.7 ± 5.38	<0.001	28.1 ± 8.87	27.5 ± 7.63	0.694	31.3 ± 6.64	23.7 ± 8.18	<0.001
AST (U/L)	29.8 ± 11.2	21.0 ± 7.02	<0.001	28.6 ± 12.1	24.8 ± 8.92	0.069	33.2 ± 10.8	19.4 ± 3.74	<0.001
Tbil (umol/L)	14.7 ± 7.24	15.4 ± 7.36	0.661	15.6 ± 8.54	14.2 ± 5.45	0.287	15.7 ± 7.52	14.1 ± 6.90	0.248
ALB (g/L)	44.5 ± 4.96	44.1 ± 5.74	0.690	43.8 ± 6.00	45.0 ± 4.15	0.228	43.1 ± 6.08	45.8 ± 3.58	0.007
GGT (U/L)	24.3 ± 22.6	19.0 ± 14.1	0.134	23.2 ± 20.6	21.7 ± 20.0	0.698	26.9 ± 22.9	17.4 ± 15.3	0.012
ALP (U/L)	69.4 ± 21.3	69.1 ± 26.0	0.947	69.0 ± 23.3	69.7 ± 22.7	0.876	71.8 ± 23.5	66.4 ± 22.0	0.223
HBV DNA (Log_10_ IU/mL)	6.90 ± 1.26	6.99 ± 1.27	0.725	5.85 ± 0.67	8.13 ± 0.27	<0.001	6.64 ± 1.16	7.26 ± 1.30	0.012
HBsAg (Log_10_ IU/mL)	3.82 ± 0.81	4.16 ± 0.66	0.022	3.41 ± 0.57	4.52 ± 0.49	<0.001	3.57 ± 0.73	4.35 ± 0.59	<0.001
HBeAg positive (%)	53 (74.6%)	31 (83.8%)	0.401	33 (57.9%)	51 (100%)	<0.001	40 (59%)	44 (88%)	0.032
PLT (×10^9^/L)	206 ± 62.8	207 ± 61.3	0.945	200 ± 63.6	213 ± 60.1	0.277	177 ± 62.8	241 ± 39.8	<0.001
TCHO (mmol/L)	4.66 ± 0.87	4.34 ± 0.88	0.070	4.41 ± 0.94	4.71 ± 0.79	0.069	4.48 ± 0.98	4.63 ± 0.76	0.391
TG (mmol/L)	1.04 ± 0.48	0.88 ± 0.37	0.060	0.96 ± 0.45	1.01 ± 0.45	0.611	1.03 ± 0.46	0.94 ± 0.44	0.290
Cr (umol/L)	61.4 ± 12.2	69.1 ± 16.1	0.013	62.6 ± 13.6	65.7 ± 14.5	0.248	63.4 ± 13.9	64.9 ± 14.4	0.574
Glu (mmol/L)	5.59 ± 0.76	5.43 ± 0.49	0.187	5.61 ± 0.77	5.44 ± 0.56	0.195	5.62 ± 0.80	5.43 ± 0.51	0.134
INR	1.13 ± 0.18	1.11 ± 0.10	0.381	1.15 ± 0.20	1.10 ± 0.08	0.098	1.17 ± 0.20	1.08 ± 0.06	0.003
AFP (ng/ml)	5.27 ± 8.51	4.64 ± 8.05	0.707	4.80 ± 6.93	5.33 ± 9.71	0.745	6.84 ± 11.0	2.97 ± 1.63	0.010
CEA	2.19 ± 1.12	2.25 ± 0.84	0.737	2.18 ± 1.14	2.25 ± 0.89	0.706	2.38 ± 1.26	2.02 ± 0.62	0.055
LSM (kPa)	9.76 ± 8.83	8.92 ± 6.35	0.576	10.5 ± 8.35	8.36 ± 7.63	0.174	11.9 ± 9.82	6.61 ± 3.69	<0.001
APRI score	0.55 (0.61)	0.36 (0.30)	0.035	0.54 (0.59)	0.43 (0.45)	0.269	0.70 ± 0.65	0.23 ± 0.04	<0.001
MASLD (%)	12 (16.9%)	4 (10.8%)	0.575	11 (19.3%)	5 (9.80%)	0.265	10 (17.2%)	6 (12%)	0.622
Ascites (%)	3 (4.23%)	3 (8.11%)	0.410	5 (8.77%)	1 (1.96%)	0.210	6 (10.3%)	0 (0.00%)	0.029
Splenomegaly (%)	12 (16.9%)	5 (13.5%)	0.857	12 (21.1%)	5 (9.80%)	0.181	13 (22.4%)	4 (8%)	0.074
Drug_type ETV	18 (25.4%)	11 (29.7%)	0.44	19 (33.3%)	10 (19.6%)	0.269	17 (29.3%)	12 (24.0%)	0.807
TDF	17 (23.9%)	5 (13.6%)		11 (19.3%)	11 (21.6%)		11 (19.0%)	11 (22.0%)	
TAF	36 (50.7%)	21 (56.8%)		27 (47.4%)	30 (58.8%)		30 (51.7%)	27 (54.0%)	

AFP, alpha-fetoprotein; ALB, albumin; ALP, alkaline phosphatase; ALT, alanine aminotransferase; AST, aspartate aminotransferase; APRI: Aspartate Aminotransferase to Platelet Ratio Index; CEA, carcinoembryonic antigen; Cr, creatinine; GGT, gamma-glutamyl transferase; Glu, glucose; HBeAg, hepatitis B e antigen; HBsAg, hepatitis B surface antigen; INR, international normalized ratio; LSM, liver stiffness measurement; MASLD, metabolic dysfunction-associated steatotic liver disease; PLT, platelets; TCHO, total cholesterol; TG, triglycerides; Tbil, total bilirubin.

Further analysis showed that the CVR rates at week 48 were comparable between the HNA subgroup and the MAG groups (56.34% (40/71) vs 55.17% (80/145), *p* = 0.99) (**Figure 3C**). Kaplan-Meier analysis demonstrated a similar likelihood of achieving a CVR at week 48 between the two groups (*p* = 0.31) (**Figure 3D**). Additionally, the mean HBV DNA levels (1.45 vs 1.45 log_10_ IU/mL, *p* = 1) and HBeAg seroconversion rates (1.89% (1/53) vs 6.48% (7/108), *p* = 0.27) were comparable between the HNA and MAG group at week 48 (**Figures 3E, F**).

#### 3.5.2 Stratified analysis by HBV DNA levels

**Figure 4:**
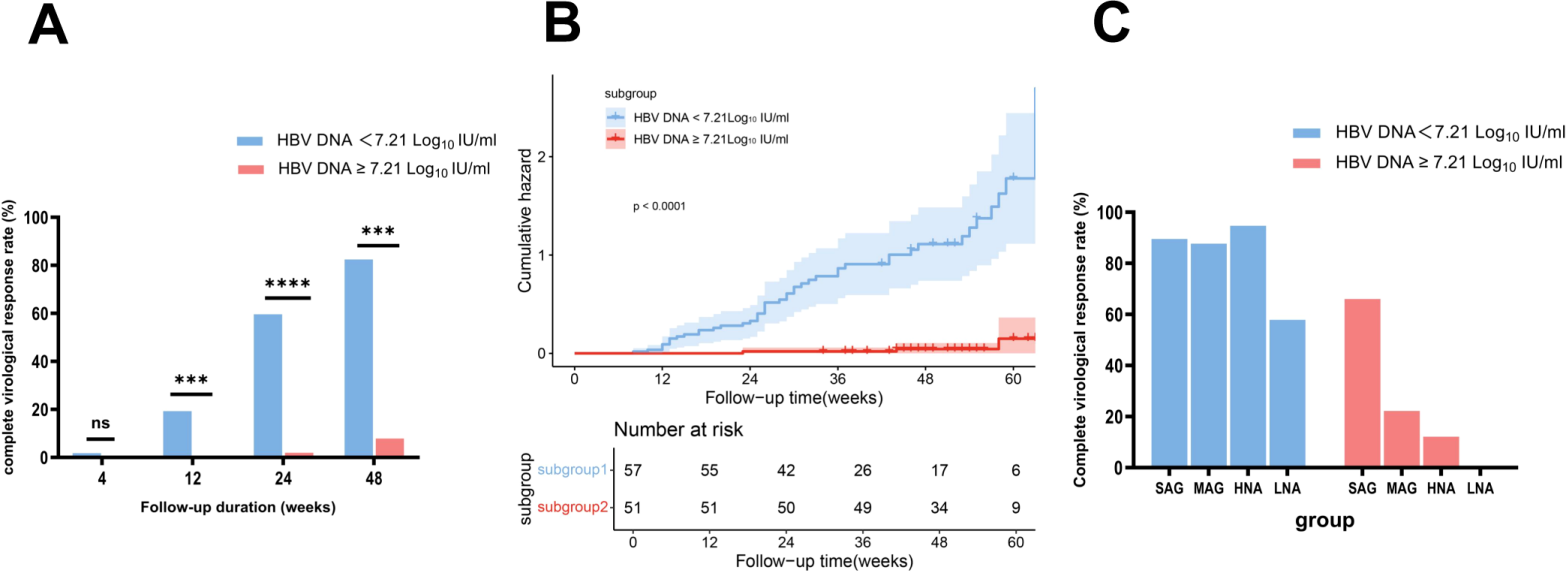
Comparison of complete virological response rates between the subgroups stratified by HBV DNA and ALT levels, with Kaplan-Meier analysis. **(A)** Comparison of complete virological response rates between the N-L-HBV DNA and N-H-HBV DNA subgroups. **(B)** Kaplan-Meier survival analysis showed a higher likelihood of achieving a complete virological response at week 48 in the N-L-HBV DNA subgroup compared to the N-H-HBV DNA subgroup. **(C)** Description of complete virological response rates in the N-L-HBV DNA and N-H-HBV DNA subgroups, stratified by ALT levels. HNA, high-normal ALT subgroup; LNA, low-normal ALT subgroup; MAG, mildly elevated ALT group; N-H-HBV DNA, high-HBV DNA subgroup in the normal ALT group; N-L-HBV DNA, low-HBV DNA subgroup in the normal ALT group; ns, no statistically significant difference; SAG, significantly elevated ALT group. ***p<0.001, ****p<0.0001.

First, the optimal baseline HBV DNA cutoff value for predicting a CVR at week 48 was determined to be 7.21 Log_10_ IU/mL through ROC curve analysis (Youden index 1.75, sensitivity 0.92, specificity 0.82) (**Supplementary Figure 2A**). Based on this cutoff, patients in the NG group were further subdivided into the N-L-HBV subgroup (HBV DNA < 7.21 Log_10_ IU/mL, n = 57) and N-H-HBV subgroup (HBV DNA ≥ 7.21 Log_10_ IU/mL, n = 51). The baseline characteristics are shown in **Table 2**. At week 48, the CVR rate was significantly higher in the N-L-HBV subgroup compared to the N-H-HBV subgroup (82.46% (47/57) vs 7.84% (4/51), *p* < 0.0001) (**Figure 4A**). Kaplan-Meier analysis indicated that the N-L-HBV subgroup had a higher likelihood of achieving CVR at week 48 (*p* < 0.0001) (**Figure 4B**).

In the N-H-HBV subgroup, no CVR (0/18) was observed in the LNA subgroup, whereas the CVR rate in the HNA subgroup was 12.12% (4/33), which was comparable to that in the MAG group (22.22% (16/72)), but lower than that in the SAG group (66.06% (109/165)). In the N-L-HBV group, the CVR rate in the LNA subgroup was 57.89% (11/19), while the CVR rate in the HNA subgroup was 94.74% (36/38), comparable to that in the MAG (87.67% (64/73)) and SAG group (89.57% (103/115)) (**Figure 4C**).

#### 3.5.3 Stratified analysis by APRI score

**Figure 5:**
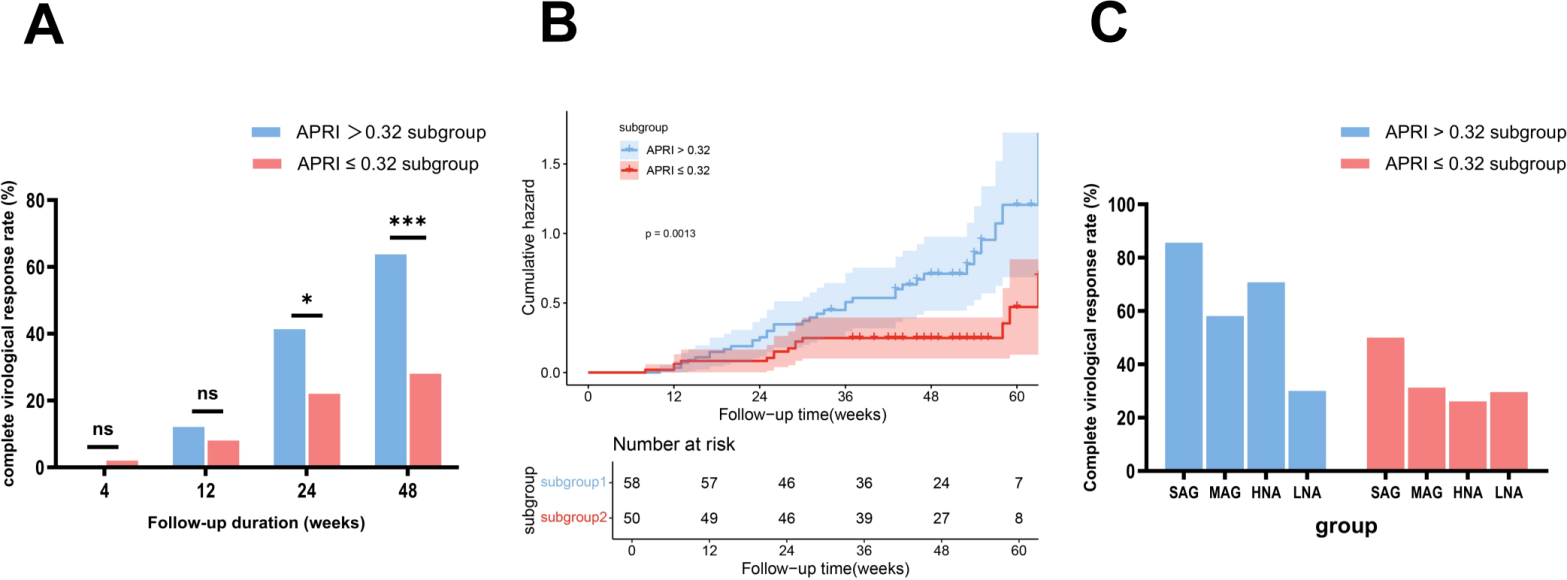
Comparison of complete virological response rates between the subgroups stratified by APRI scores and ALT levels, with Kaplan-Meier analysis. **(A)** Comparison of complete virological response rates between the N-H-APRI and N-L-APRI subgroups. **(B)** Kaplan-Meier survival analysis showed a higher likelihood of achieving a complete virological response in the N-H-APRI subgroup compared to the N-L-APRI subgroup. **(C)** Description of complete virological response rates in the N-H-APRI and N-L-APRI subgroups, stratified by ALT levels. HNA, high-normal ALT subgroup; LNA, low-normal ALT subgroup; MAG, mildly elevated ALT group; N-H-APRI, high-Aspartate Aminotransferase to Platelet Ratio Index score-subgroup in the normal ALT group; N-L-APRI, low-Aspartate Aminotransferase to Platelet Ratio Index score-subgroup in the normal ALT group; ns, no statistically significant difference; SAG, significantly elevated ALT group. *p<0.05, ***p<0.001.

The optimal baseline APRI cutoff value was determined to be 0.32 (Youden index 1.36, sensitivity 0.73, specificity 0.63) (**Supplementary Figure 2B**). Patients in the NG group were divided into the high-APRI subgroup (N-H-APRI, n=58) and the low-APRI subgroup (N-L-APRI, n=50). **Table 2** presents the baseline characteristics. At week 48, the CVR rate was significantly higher in the N-H-APRI subgroup than in the N-L-APRI subgroup (63.79% (37/58) vs 28% (14/50), *p* = 0.0002) (**Figure 5A**). Kaplan-Meier analysis showed a higher likelihood of achieving a CVR in the N-H-APRI subgroup (*p* = 0.0013) (**Figure 5B**).

In the N-H-APRI subgroup, the CVR rates in the LNA, HNA, MAG, and SAG groups was 30% (3/10), 70.83% (34/48), 58.14% (75/129), and 85.61% (238/278), respectively. In the N-L-APRI subgroup, the CVR rate in the LNA, HNA, MAG, and SAG groups was 29.63% (8/27), 26.09% (6/23), 31.25% (5/16), and 50% (1/2) respectively (**Figure 5C**).

### 3.6 Factors associated with virological response in the normal ALT group

Firstly, the optimal baseline HBsAg cutoff was 4.35 Log_10_ IU/mL (Youden index 1.66, sensitivity 0.98, specificity 0.68) (**Supplementary Figure 2C**). Additional optimal cutoffs were identified for HBV DNA decline: 3.49 Log_10_ IU/mL at 12 weeks (Youden index 1.38, sensitivity 0.87, specificity 0.51) and 4.77 Log_10_ IU/mL at 24 weeks (Youden index 1.21, sensitivity 0.69, specificity 0.51) (**Supplementary Figures 2D, E**).

Then, the univariate Cox regression analysis included various factors, such as age, gender, drinking habits, diabetes, MASLD status, baseline ALT, HBV DNA, HBsAg levels, HBeAg status, HBV DNA decline at weeks 12 and 24, APRI score, total cholesterol, creatinine, glucose, AFP, and others. **Table 3** identifies the following as negative predictors of CVR: baseline ALT < 30 U/L for males or < 19 U/L for females, HBV DNA > 7.21 Log_10_ IU/mL, HBsAg > 4.35 Log_10_ IU/mL, HBeAg positive status, APRI < 0.32, and HBV DNA decline < 3.49 Log_10_ IU/mL at 12 weeks (*p* < 0.05, HR < 1). Conversely, diabetes and baseline glucose levels were positive predictors (*p* < 0.05, HR > 1). Although age showed statistical significance (*p* < 0.05), HR = 1 may indicate no impact on outcomes. These factors were included in the multivariate analysis. Multivariate analysis confirmed that baseline ALT < 30 U/L for males or <19 U/L for females (HR=0.27, 95% CI: 0.11-0.67, *p*=0.005), HBV DNA > 7.21 Log_10_ IU/mL (HR=0.12, 95% CI: 0.03-0.52, *p*=0.005), HBeAg positive status (HR=0.29, 95% CI: 0.13-0.63, *p*=0.002), APRI < 0.32 (HR=0.39, 95% CI: 0.17-0.91, *p*=0.03), and HBV DNA decline < 3.49 Log_10_ IU/mL at 12 weeks (HR=0.33, 95% CI: 0.12-0.93, p=0.04) were independent negative predictors.

**Table 3:** Univariate and multivariate cox regression analysis of predictors for virological response in patients with ALT ≤ 40 U/L.

	Univariable		Multivariable	
	*p*	HR (95% CI)	*p*	HR (95% CI)
Age	0.0041	1 (1-1.1)	0.758	0.99 (0.96-1.03)
Gender	0.14	0.64 (0.35-1.2)		
Hypertension	0.058	2.5 (0.97-6.2)		
Diabetes	0.043	4.4 (1-18)	0.317	0.35 (0.04-2.74)
Alcohol habit	0.25	0.31 (0.041-2.3)		
ALT < 30 U/L for male or < 19 U/L for female	0.0083	0.4 (0.21-0.79)	0.005	0.27 (0.11-0.67)
Baseline HBV DNA > 7.21 Log_10_ IU/mL	<0.0001	0.061 (0.022-0.17)	0.005	0.12 (0.02-0.52)
Baseline HBsAg > 4.35 Log_10_ IU/mL	0.0002	0.024 (0.003-0.17)	0.152	0.18 (0.017-1.88)
Baseline HBeAg positive	<0.0001	0.13 (0.074-0.23)	0.002	0.29 (0.13-0.63)
HBV DNA decline < 3.49 Log_10_ IU/mL at 12 weeks of antiviral treatment	0.0029	0.24 (0.093-0.61)	0.035	0.39 (0.17-0.91)
HBV DNA decline < 4.77 Log_10_ IU/mL at 24 weeks of antiviral treatment	0.11	1.8 (0.88-3.6)		
PLT	0.46	1 (1-1)		
TCHO	0.36	1.2 (0.84-1.6)		
TG	0.52	1.2 (0.66-2.3)		
Cr	0.29	0.99 (0.97-1)		
Glu	0.048	1.4 (1-2)	0.396	1.24 (0.75-2.04)
INR	0.81	0.8 (0.13-5)		
AFP	0.66	0.99 (0.96-1)		
CEA	0.57	0.92 (0.7-1.2)		
LSM	0.83	1 (0.96-1)		
APRI score < 0.32	0.002	0.38 (0.2-0.7)	0.04	0.33 (0.12-0.93)
MASLD	0.07	1.9 (0.95-3.8)		
Ascites	0.34	0.5 (0.12-2.1)		
Splenomegaly	0.58	0.81 (0.38-1.7)		
Drug_type ETV	Ref	Ref		
TDF	0.54	0.82 (0.43-1.56)		
TAF	0.78	0.89 (0.39-2.01)		

AFP, alpha-fetoprotein; ALT, alanine aminotransferase; APRI, Aspartate Aminotransferase to platelet ratio index; CEA, carcinoembryonic antigen; Cr, creatinine; Glu, glucose; HBeAg, hepatitis B e antigen; HBsAg, hepatitis B surface antigen; INR, international normalized ratio; LSM, liver stiffness measurement; MASLD, metabolic dysfunction-associated steatotic liver disease; PLT, platelets; TCHO, total cholesterol; TG, triglyceride.

### 3.7 A predictive model for CVR in normal ALT group

**Figure 6:**
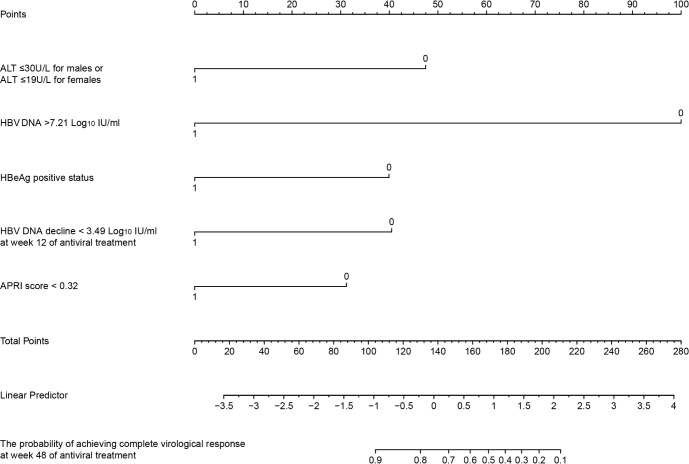
Nomogram for predicting the probability of achieving complete virological response at 48 weeks of antiviral treatment in treatment-naïve patients with ALT ≤ 40 U/L. ALT, alanine aminotransferase; APRI, Aspartate Aminotransferase to Platelet Ratio Index score; HBeAg, hepatitis B e antigen.

We developed the Predictor of Antiviral Therapy for HBV (Pra-HBV) model based on Cox regression to enhance clinical applicability. This model includes the following predictors: baseline ALT (< 30 U/L for males or < 19 U/L for females), HBV DNA > 7.21 Log_10_ IU/mL, HBeAg positive status, APRI < 0.32, and HBV DNA decline < 3.49 Log_10_ IU/mL at 12 weeks. These factors may predict the probability of achieving a CVR at week 48 in patients with ALT ≤ 40 U/L early in treatment (**Figure 6**). The model’s C-index was 0.86, and the calibration plot showed good agreement between the predicted and observed values (**Supplementary Figure 3**).

## 4 Discussion

Serum ALT levels reflect the degree of liver inflammation and the host immune response to HBV infection. ALT is recommended by multiple guidelines as a crucial non-invasive indicator for assessing the immune stage in chronic HBV infection. Elevated ALT levels are typically associated with liver inflammation or fibrosis. However, liver inflammation and immune infiltrates can still occur in patients with normal ALT. One study found that 28.7% of CHB patients with ALT <40 U/L and detectable HBV DNA had significant inflammation on liver biopsy (10). Similarly, another study analyzing liver biopsies from 1,043 CHB patients with normal ALT levels reported that over 70% had significant histologic lesions (11). These findings indicate that some patients with normal ALT still have liver inflammation and immune infiltrates, which are key contributors to disease progression, cirrhosis, and HCC. In patients with normal ALT and negative HBeAg, antiviral treatment was associated with a 76% reduction in the risk of developing liver nodules and cirrhosis compared to those who were untreated (12). Early antiviral treatment in such patients not only suppresses viral replication but also improves clinical outcomes. Therefore, several guidelines recommend initiating antiviral therapy as soon as ALT exceeds the upper limit of normal (ULN). However, in contrast to patients with elevated ALT, the evidence regarding the effect of antiviral therapy in patients with normal ALT remains limited, and the findings from existing studies are inconsistent.

Some studies have demonstrated that antiviral therapy can achieve favorable outcomes even in patients with low ALT levels, such as a meta-analysis of five studies, which showed that antiviral treatment in HBV-infected patients with immune-tolerant status and low ALT levels promoted HBV DNA suppression and HBsAg loss, but had no significant impact on HBeAg seroconversion (13). However, three of these studies set the ULN of ALT level at 40 U/L, and the overall cohort had a mean ALT level exceeding 30 U/L. This suggests that some immune-active patients were included. Therefore, this study was unable to accurately represent the virological response of patients with genuinely low ALT levels. Other studies by Hu and Wei found no significant differences in virological response rates between patients with normal and mildly elevated ALT (14, 15). In contrast, Zhao’s study reported higher virological response rates in CHB patients with normal ALT than in those with mildly elevated ALT at weeks 24 and 48 of antiviral therapy, though the rates became similar by week 72 (16). Notably, patients with normal ALT levels in Zhao’s study had lower HBV DNA, HBeAg, and HBsAg at baseline compared to those with mildly elevated ALT. However, these indicators are critical factors influencing virological response, as demonstrated in our study. Based on this background, we utilized IPTW to rigorously match all virological indicators and confounding variables at baseline, ensuring meticulous adjustments for a balanced and unbiased comparison. Our study concluded that patients with normal ALT levels had significantly lower CVR rates and HBeAg seroconversion rates at week 48. As for minimizing the influence of confounding variables, our findings could more precisely reflect clinical practice, providing a more reliable and robust assessment of antiviral treatment outcomes, which may be directly applied to real-world practice.

The latest guidelines for the prevention and treatment of CHB in China (17) indicate that individuals with positive HBV DNA and abnormal ALT should receive active antiviral therapy. However, in most hospitals in China, the normal ALT value is set at 40 U/L for females or 50 U/L for males. Our team previously analyzed the relationship between ALT levels and liver biopsy severity. The results showed that when ALT was less than 0.5×ULN, 30.4% of patients had a ≥ G2 and 21.8% were ≥ S2. At ALT levels between 0.5 and 1×ULN, 36.6% were ≥ G2 and 29.3% were ≥ S2. These findings suggest that the current ULN standard may not effectively distinguish the liver status. A recent study, incorporating epidemiological changes in major liver diseases (hepatitis B, C, HIV, and alcohol use), recommended setting the upper limit of normal ALT levels at 34 U/L for men and 22 U/L for women (18). Additionally, Kang studied 1,101 treatment-naïve CHB patients with ALT < 40 U/L and liver biopsy results, finding that ALT levels of 27 U/L for males and 24 U/L for females were more accurate predictors of histological changes in the liver (19). Other studies suggest that immune-tolerant patients with ALT levels of 20-39 U/L face a higher risk of progressing to HBeAg-positive CHB than those with ALT < 20 U/L (20). According to the latest WHO guidelines, antiviral therapy is recommended for males with ALT > 30 U/L and females with ALT > 19 U/L. Therefore, in our study, stratification analysis based on baseline ALT, HBV DNA, and APRI scores was conducted to explore the underlying determinants that might affect the suboptimal outcomes in the normal ALT group. Patients in this group were divided into two subgroups: the high-normal ALT subgroup (30-40 U/L for males or 19-40 U/L for females) and the low-normal ALT subgroup (≤ 30 U/L for males or ≤ 19 U/L for females). The results showed that the CVR rate in the high-normal ALT subgroup was significantly higher than in the low-normal ALT subgroup and, most notably, comparable to that in the mildly elevated ALT group (40-80 U/L). Further subgroup analysis based on baseline HBV DNA levels and APRI scores in patients with high-normal ALT revealed that, regardless of HBV DNA or APRI levels, patients in this subgroup achieved similar virological response rates to those with the same HBV DNA or APRI levels but with mild ALT elevation. This finding strongly supports that patients with ALT levels between 30-40 U/L in males and 19-40 U/L in females could achieve better virological response at 48 weeks. These compelling results provide robust evidence supporting the latest WHO guideline recommendations, emphasizing the practicality and effectiveness of initiating antiviral therapy when ALT exceeds 30 U/L in males and 19 U/L in females in clinical decision-making. This reinforces their importance in optimizing treatment strategies for patients with normal ALT. In clinical practice, early antiviral therapy for this subset can reduce the risk of poor prognosis and increase the likelihood of achieving a complete virological response within the target time.

For patients in the low-normal ALT subgroup, those with lower HBV DNA levels also achieved a good CVR rate. However, the results became particularly striking when we focused on patients with baseline HBV DNA ≥ 7.21 Log_10_ IU/mL, none of them achieved CVR at 48 weeks. This highlights a key finding: a high viral load combined with low ALT levels might indicate an immune-tolerant state, which is less likely to respond to antiviral treatment, while those with lower HBV DNA levels were likely already in the immune-active state and therefore could achieve a good viral response. Additionally, we found that patients with lower APRI scores in the normal ALT group had a lower CVR rate. These findings suggest that low ALT, high HBV DNA, and low APRI scores are negative factors for virological response. Patients exhibiting these characteristics may be in an immune tolerance state. In this phase, HBV evades host immune surveillance by suppressing interferon and pro-inflammatory cytokine production, thus preventing innate immune system activation and enabling persistent viral infection (21). Furthermore, in the immune-tolerant state, HBV-specific CD8+ T-cell responses are impaired in both quantity and quality (22). These reasons explain why patients with low ALT, high HBV DNA, and low APRI scores have poor virological responses.

Based on these findings, we developed a predictive model, Pra-HBV, which integrates critical baseline factors, including baseline ALT < 30 U/L for males or < 19 U/L for females, HBV DNA > 7.21 Log_10_ IU/mL, HBeAg positive status, APRI score < 0.32, and HBV DNA decline < 3.49 Log_10_ IU/mL at 12 weeks. This model incorporates a manageable number of key factors and serves as a helpful tool for predicting the 48-week complete virological response rate. It can help guide early adjustments to antiviral regimens, improving the likelihood of achieving optimal treatment outcomes. The Pra-HBV model had exceptional predictive accuracy in our study, with a C-index of 0.86, underscoring its ability to forecast virological response. Pra-HBV can assist in personalizing treatment strategies and properly managing patients with normal ALT levels during the early stage of antiviral treatment, particularly those with low ALT levels, high viral loads, and low APRI scores. However, external validation with larger cohorts is crucial to confirm its reliability and generalizability, ensuring that this model can ultimately be applied on a broader scale to improve patient outcomes across diverse populations.

The study also has certain limitations. Owing to the small sample size of patients with ALT levels below 40 U/L, this might affect the subgroup analyses. Future studies will require a larger sample size to validate our findings and more thoroughly confirm the model’s efficacy and reliability. Furthermore, because of various factors, the number of patients followed up for 48-96 weeks was limited, and the complete virological response rate at 96 weeks was not included in our analysis. It is essential to evaluate whether extending the treatment duration can significantly improve the complete virological response rates. Considering factors such as economic constraints, it may be important to explore whether a “watchful waiting” approach could be a viable option for certain patients. Future research could construct a prospective cohort by collecting samples from patients with low ALT and high DNA levels at baseline. Analyzing their immune environment will provide insights into their immune status at the start of antiviral treatment, aiding in more accurate predictions of treatment outcomes and enhancing the reliability of our research.

In conclusion, we found that patients with ALT levels of 30-40 U/L for males and 19-40 U/L for females achieve good CVR rates, similar to those with elevated ALT, regardless of their HBV DNA levels and APRI scores. However, patients with low ALT and APRI scores but high viral load are unlikely to achieve antiviral efficacy with mono-NAs. The Pra-HBV model can be used to predict these patients’ virological response at the start of antiviral treatment. Early combination therapy or alternative strategies could be considered to achieve CVR, and we also hope that the development of new clinical drugs will further enhance treatment efficacy for this patient group.

## Data Availability

The raw data supporting the conclusions of this article will be made available by the authors, without undue reservation.
