# Supplementary material for: Virological response and predictive factors for antiviral treatment in chronic HBV-related liver disease with low ALT and high HBV DNA
Source: Front Immunol. 2025 Feb 26;16:1556547. doi: 10.3389/fimmu.2025.1556547 (PMC11897475; doi:10.3389/fimmu.2025.1556547)
Supplement: Supplementary file 1 [file DataSheet1.docx]

Supplementary Material

## 1. Supplementary Figure


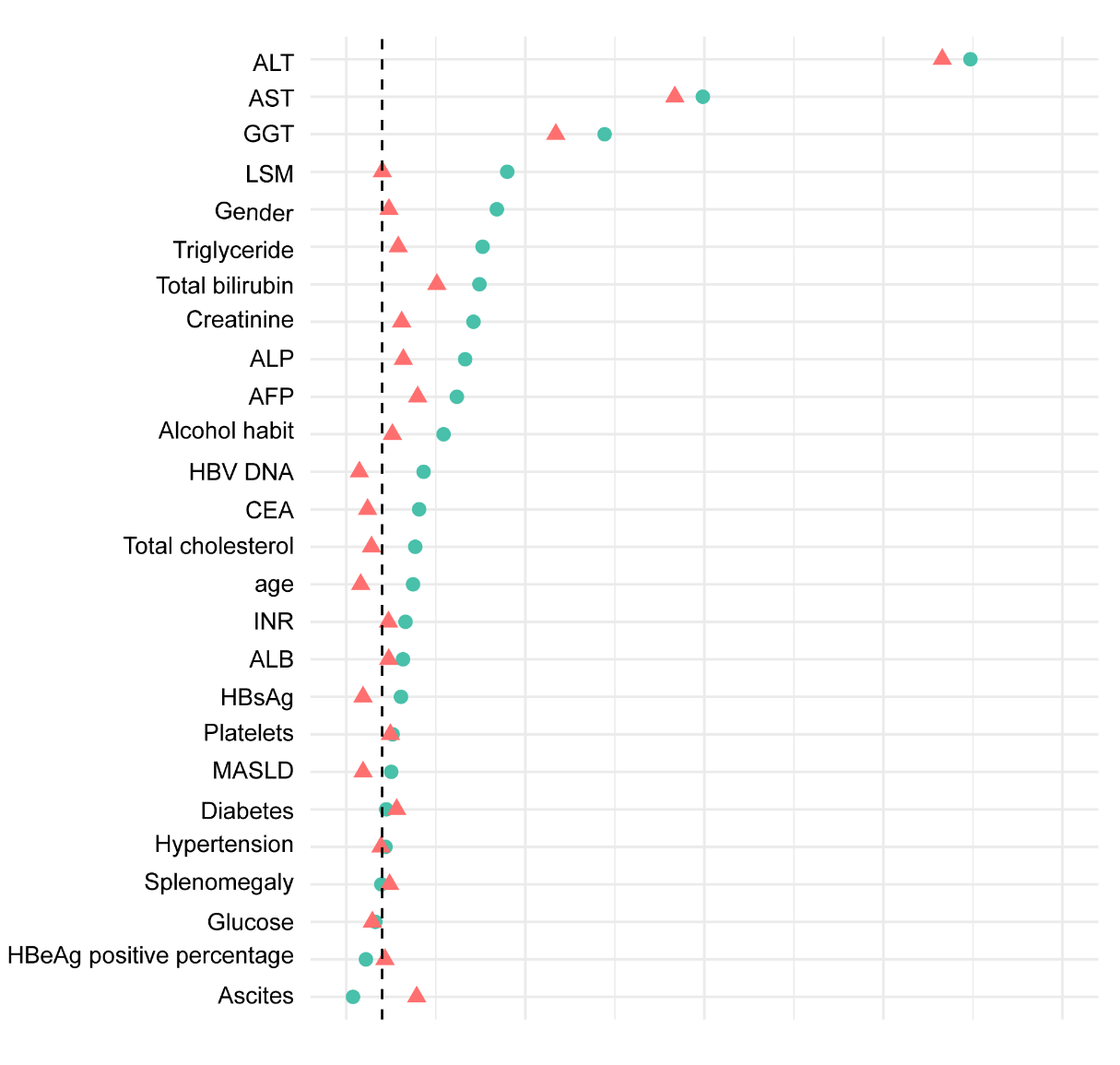


**Supplementary Figure 1.** Average standardized mean difference of baseline indicators' balance among three groups after IPTW. Abbreviations: AFP, alpha-fetoprotein; ALB, albumin; ALP, alkaline phosphatase; ALT, alanine aminotransferase; AST, aspartate aminotransferase; CEA, carcinoembryonic antigen; GGT, gamma-glutamyl transferase; HBeAg, hepatitis B e antigen; HBsAg, hepatitis B surface antigen; INR, international normalized ratio; LSM, liver stiffness measurement; MASLD: metabolic dysfunction-associated steatotic liver disease.


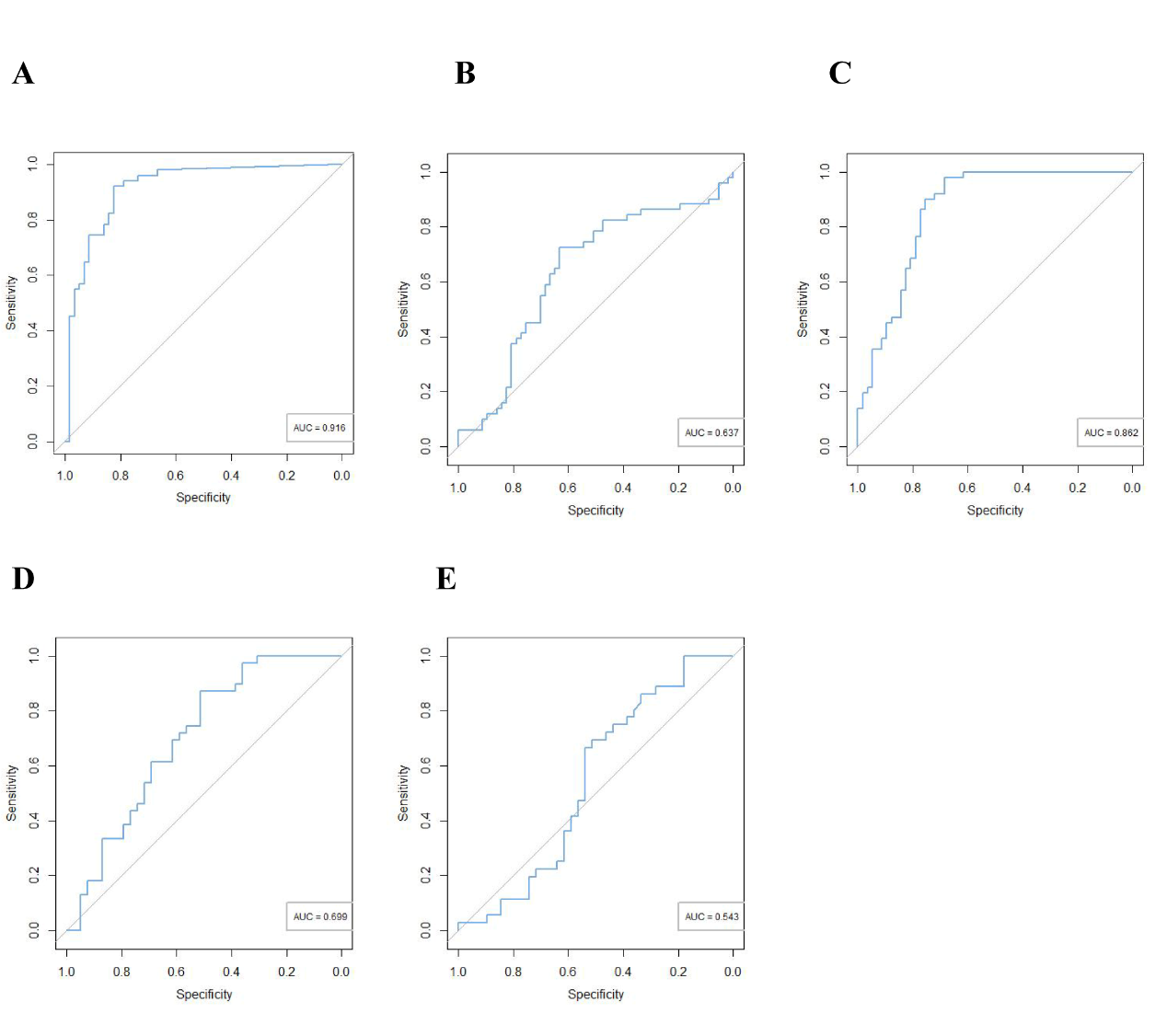


**Supplementary Figure 2.** ROC curve for baseline Log_10_ HBV DNA, APRI scores, Log_10_ HBsAg, Log_10_ HBV DNA decline at week 12, Log_10_ HBV DNA decline at week 24 of antiviral treatment respectively.


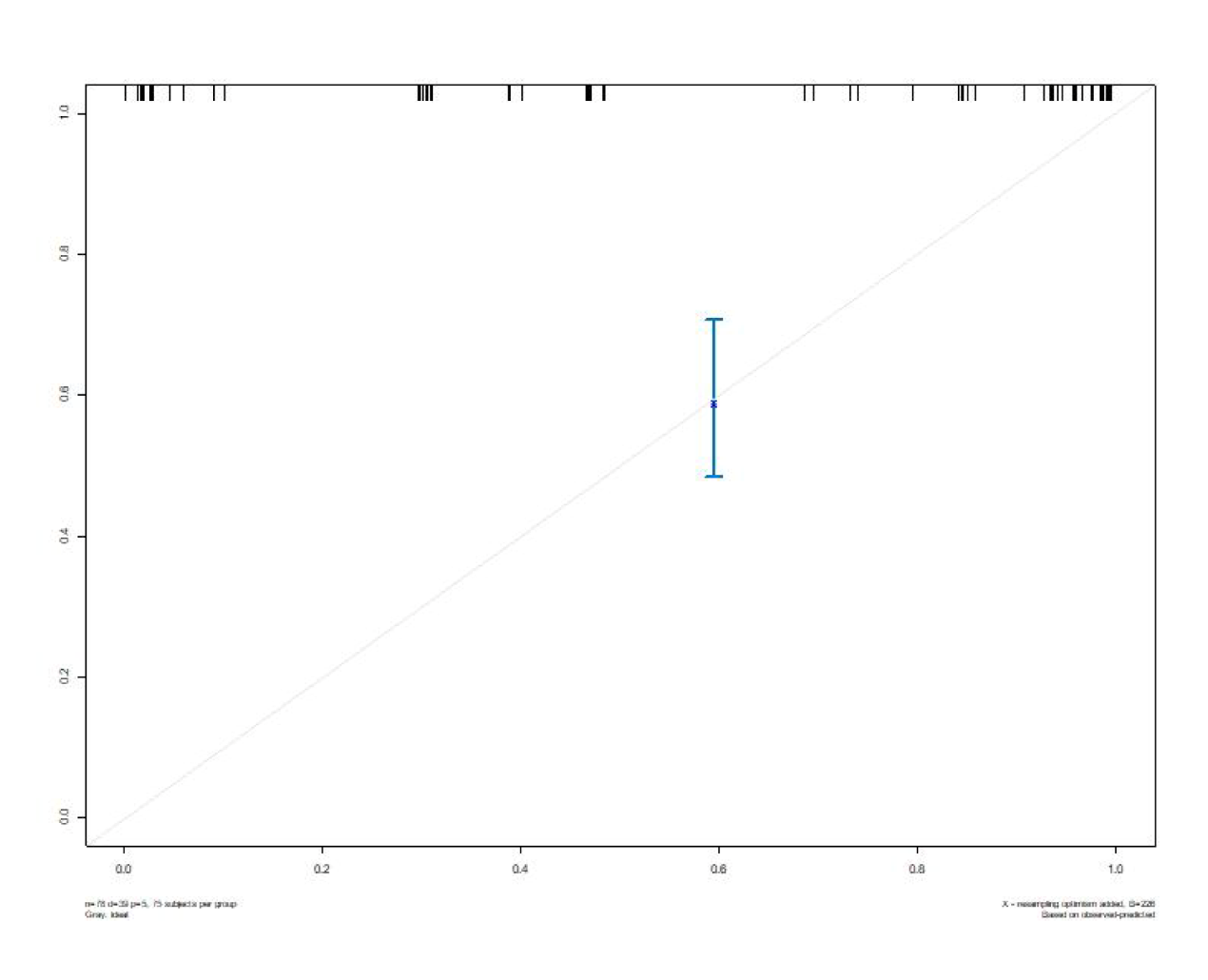


**Supplementary Figure 3.** Calibration plots of predicting the probability of achieving complete virological response at week 48 of antiviral treatment in treatment-naïve patients with ALT ≤ 40 U/L based on Cox regression.

## 2. Supplementary Table

**Supplementary Table 1** Subgroup Classification and Names within the ALT ≤40 U/L group

|  | Levels | Names | Abbreviations |
| --- | --- | --- | --- |
| ALT levels (U/L) | 30-40 for male, 19-40 for female | high-normal ALT subgroup | HNA |
|  | ＜30 for male, ＜19 for female | low-normal ALT subgroup | LNA |
| HBV DNA levels (IU/ml) | ＞ 7.21 Log_10_ IU/ml | high-HBV DNA subgroup | N-H-HBV |
|  | ＜ 7.21 Log_10_ IU/ml | low-HBV DNA subgroup | N-L-HBV |
| APRI scores | ＞ 0.32 | high-APRI subgroup | N-H-APRI |
|  | ＜ 0.32 | low-APRI subgroup | N-L-APRI |
